# Wake‐up strokes are linked to obstructive sleep apnea and worse early functional outcome

**DOI:** 10.1002/brb3.2284

**Published:** 2021-07-21

**Authors:** Tuuli‐Maria Haula, Juha Puustinen, Mari Takala, Anu Holm

**Affiliations:** ^1^ Unit of Neurology Satakunta Hospital District Clinical Neurosciences University of Turku Pori Finland; ^2^ Unit of Neurology Satakunta Hospital District Social Security Centre of Pori City of Pori Clinical Pharmacy Group University of Helsinki Pori Finland; ^3^ Unit of Clinical Neurophysiology Satakunta Hospital District Pori Finland; ^4^ Unit of Clinical Neurophysiology Satakunta Hospital District Faculty of Health and Welfare Satakunta University of Applied Sciences Pori Finland

**Keywords:** ischemic stroke, obstructive sleep apnea, wake‐up stroke

## Abstract

**Background and Aims:**

Presence of sleep‐disordered breathing (SDB) and especially obstructive sleep apnea (OSA) is a known risk factor for ischemic stroke. Additionally, SDB effects negatively on recovery after stroke. Up to one fourth of strokes are present on awakening. The link between OSA and wake‐up stroke (WUS) has been suggested. We aim to determine the association between OSA and WUS in a Finnish stroke unit cohort.

**Material and Methods:**

An observational prospective longitudinal study consisted of 95 TIA (transient ischemic attack) and mild to moderate stroke patients referred to a Stroke Unit in Finland. Respiratory polygraphy was performed within 72 h of hospital admission. Patients were classified into WUS and non‐WUS, and functional outcome measures (mRS, rehabilitation, hospitalization time) were collected. Functional outcomes and prevalence of OSA were compared between non‐WUS and WUS.

**Results:**

OSA (AHI > 15/h) was more frequent among WUS than non‐WUS (71% and 36%, respectively, *p* = 0.009). Functional outcome measured with mRS was worse in patients with WUS than non‐WUS on registration day and at hospital discharge (*p* = 0.001). Need for rehabilitation in WUS was 43% of cases compared to 23% of non‐WUS (*p* = 0.067). Hospitalization time was longer (5–15days) in 55% of WUS and 41% of non‐WUS patients (*p* = 0.261).

**Conclusion:**

Moderate‐to‐severe OSA is related to WUS compared to non‐WUS. In addition, WUS have worse short‐term outcomes measured in mRS. Further studies are needed to determine if OSA is causally linked to WUS.

## Graphical abstract

The aim of this observational longitudinal study is to determine the relationship between WUS and OSA .We conclude that the proportion of moderate to severe OSA is higher stroke and TIA patients with WUS compared to patients with non‐WUS. WUS have worse short‐term outcomes measured in mRS. Further studies are needed to determine if OSA is causally linked to WUS.

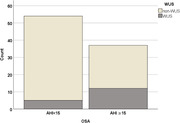

## 1 INTRODUCTION

Sleep‐disordered breathing (SDB) is frequent among patients with TIA (transient ischemic attack) or ischemic stroke. Several studies have estimated the prevalence of SDB between 50% to even as high as 90% depending on the definition (Bassetti & Aldrich, 1999; Bassetti et al., 1996; Hermann & Bassetti, 2016; Hermann & Bassetti, 2009; Huhtakangas et al., 2017; Sahlin et al., 2008). In stroke care, SDB and especially the most common condition, obstructive sleep apnea (OSA) is a preceding condition and a known risk factor for ischemic stroke (European Stroke Organisation (ESO) Executive Committee, 2008; Yaggi et al., 2005; Young et al., 2002). Wake‐up stroke (WUS) is an ischemic event that occurs during nocturnal sleep meaning the patient is asymptomatic when fell asleep and wakes up with neurological deficits. Studies have shown the prevalence of WUS to be 20–25% of all stroke (Chaturvedi et al., 1999; Elliott, 1998; Lago et al., 1998; Wroe et al., 1992). The circadian pattern of stroke onset is observed by previous studies. There is an increased risk of TIA and ischemic strokes during early and late morning hours (Elliott, 1998; Kelly‐Hayes et al., 1995; Marler et al., 1989). Explanations for WUS might be recurrent hypoxemia, variable blood pressure, platelet aggregation, increased cardiac arrhythmias, cerebral hypoperfusion, and sleep apnea. (Andreotti et al., 1988; Andrews et al., 1996; Bassetti & Aldrich, 1999; Chin et al., 1996; Millar‐Craig et al., 1978; Netzer et al., 1998) The relationships between WUS and OSA has been proposed by several studies (Bassetti et al., 2006; Hsieh et al., 2012; Iranzo et al., 2002; Mohammad et al., 2019) and WUS has been linked to certain stroke subtypes like micro‐ and macroangiopathic strokes. (Bassetti & Aldrich, 1999; Bassetti et al., 2006; Lago et al., 1998; Spengos et al., 2005; Tanimoto et al., 2014) Further, the same stroke subtypes are also seen in patients with OSA. (Bassetti et al., 2006; Bassetti; Harbison, 2002)

The aim of this observational longitudinal study is to determine the relationship between WUS and OSA in a Finnish stroke unit cohort which includes mainly minor strokes and TIA patients. This is the first study on this topic in a Finnish stroke population. The hypothesis was that OSA is linked to WUS and short‐term outcome is worse in WUSs.

## 2 MATERIAL AND METHODS

### 2.1 Study population

This study is a part of a larger research of SDB in Finnish stroke patients in Satakunta Hospital District, Finland. Patients were recruited from the Stroke Unit between March 2013 and November 2016. We included 102 patients with first ever acute ischemic stroke or TIA who were able to give informed consent and had less than 72 h of hospital admission. Data from five patients was not analyzed due to technical problems or devices were detached. Two patients not fulfilling inclusion criteria were excluded (diagnosis intracerebral haemorrhage and glioma). Few patients reported later that they had fluctuating symptoms or prior symptoms within the preceding. Additionally, the patients were not allowed to have previous strokes, diagnosis, or treatment for sleep apnea. Moreover, the patients had to be conscious and not suffer from disabling aphasia to give written informed consent. The diagnosis of stroke or TIA was confirmed by Neurologist based on a history of a sudden onset of a neurologic deficit, clinical examination, and brain imaging. The registrations were conducted by Scientist during weekdays, from Monday to Thursday, due to practical reasons. Despite of that, patients admitted to Stroke Unit during weekend could be recruited and registered because of the inclusion criteria.

A detailed medical history and neurological examinations were done according to the hospital protocol by Neurologist. Following demographic data was collected by the Scientist: Sex, age, BMI (body mass index), smoking, previous diseases, marital status, education, and occupation. The severity of the stroke was estimated by the National Institute of Health Stroke Scale (NIHSS) (Brott et al., 1989) and disability was evaluated using Barthel Index scale (BI) (Mahoney & Barthel, 1965) on registration day. Basic Nordic Sleep Questionnaire (Partinen & Gislason, 1995) were filled before registration. Computer tomography scan (CT) and on selected patients, magnetic resonance imaging were performed. All routine laboratory tests, ECG and chest X‐ray, and blood pressure were examined according to the normal protocol of stroke and TIA patients. The patients were divided into two groups, WUS (stroke onset during nocturnal sleep) and non‐WUS (stroke onset awake).

This research project was approved by the Ethical Board of Hospital District of Southwest Finland. Participation to the study was optional and the participant had to give their written informed consent. The caregiver or relative could not give informed consent according to the Ethics committee.

### 2.2 Assessment

All recruited patients were admitted to Stroke Unit through Emergency Room (ER) at Satakunta Hospital District. The onset of stroke was mentioned in medical reports either in ER or in Stroke Unit. Definition of WUS means that the patient is asymptomatic before going to sleep at night and wakes up with neurological deficits in the morning. The stroke subtype was categorized by TOAST classification (Adams et al., 1993) to 1) large‐artery atherosclerosis, 2) cardioembolism, 3) small vessel occlusion, 4) strokes of other determined etiology, and 5) stroke of undetermined etiology.

In addition, we evaluated the relationships between WUS and clinical outcome, measured in modified Rankin Scale, hospitalization time and need of rehabilitation. In this study, SDB was tested with respiratory polygraphy which is considered as a sufficient method for diagnosis. (Erkinjuntti et al., 2006; Goldberg, 2007; Parra et al., 1997) The participants were categorized to two classes of OSA according to the respiratory recording in acute phase (non‐OSA is AHI < 15/h and OSA is AHI > 15/h).

Outcome measures were assessed from full medical records by Neurologist blinded by apnea‐hypopnea index (AHI) severity. Disability was assessed by modified rankin scale (mRS) in each patient before hospital admission (mRSpre), on registration day (mRS0) and at hospital discharge (mRSexit). The scale was divided to four categories: mRS 0, mRS 1‐2, mRS 3‐5, and mRS 6. The first category of mRS 0 includes patients who are asymptomatic and mRS 1‐2 includes patients who have slight disability or any symptoms from stroke but are independent in activity of daily living (ADL) functions. These two categories are defined as a favorable outcome. Modified Rankin Scale 3‐5 includes patients who have higher disability meaning the patient needs help at least in some ADL functions and mRS 6 is death. These categories are defined as a non‐favorable outcome. Hospitalization time was calculated from the medical records and for the analyses, it was divided into two groups, 2–4 days (shorter hospital stay) and 5–15 days (longer hospital stay). At the Neurologic ward, the present study is conducted, 4.3 days have been the mean time spent in the hospital. Thus, longer hospitalization (5–15 days) used in this study is over the mean length of hospital stay. The information if patient needed rehabilitation was retrieved from the medical records and rehabilitation included “basic” rehabilitation in rehabilitation units of health care centers and in multidisciplinary advanced rehabilitation units.

### 2.3 Sleep measurements

All patients underwent overnight respiratory polygraphy within 48 h after admission to Stroke Unit. In this study the recordings were made with NOX T3 wireless recorder (Copyright Nox Medical). The device measures ECG with a sampling frequency of 200 Hz, airflow with nasal cannulae, oxygen saturation with plethysmography, snoring sound and breathing effort. Nocturnal respiratory recording measures respiratory events during sleep. It does not include EEG (electroencephalography).

NoxT3 respiratory polygraphy have been validated and AHI can be used in scoring apneas instead of respiratory event index that have been used in home sleep apnea testing. (Cairns et al., 2014; Wen et al., 2019; Xu et al., 2017)

The data was scored for apneas and hypopneas according to the recommendations of American Academy of Sleep Medicine (AASM 2012) and expressed as AHI. (Berry &, Brooks) The definition of apnea is at least 90% drop of airflow signal lasting at least 10 s. Hypopnea is characterized by at least 30% drop in the airflow signal for 10 s or more. A contemporary drop of 3% or more in the blood oxygenation level should be present as well for it to count as hypopnea or arousal. If simultaneous pause in breathing effort measured by abdominal and thoracic belts occur, the apnea is classified as central sleep apnea (CSA). CSA patients were not included in OSA analyses.

The recording devices were inserted in the afternoon for practical reasons, except the nasal cannulae which were placed before patient was going to sleep. The positioning of the nasal cannulae was done by the registered nurses. In the following morning, the Scientist helped the patient to take off the equipment and the data was later downloaded into the software for analysis. The analysis of the recordings was analyzed by Clinical Neurophysiologist.

### 2.4 Statistical analysis

Statistical analyses were performed using the SPSS software version 25.0. To compare stroke outcomes (mRS scale, hospitalization time, and need for rehabilitation), baseline characteristics and existence of OSA between non‐WUS and WUS groups, Mann–Whitney test was used. *P* value < 0.05 was considered as statistically significant result.

## 3 RESULTS

**FIGURE 1:**
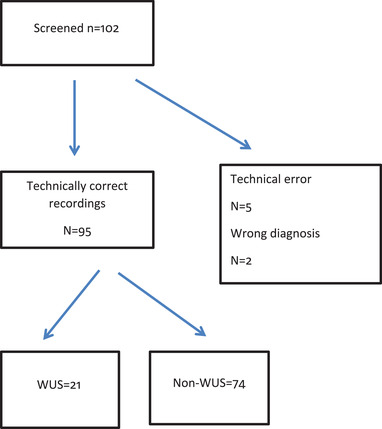
Flowchart of participants in the present study

Altogether 102 patients with acute ischemic stroke or TIA referred to the Stroke Unit were included in the present study. Five patients were not analyzed due to technical problems or devices were detached. Two patients were excluded because of wrong diagnosis (intracerebral hemorrhage and glioma) Figure 1.

Of the 95 analyzed patients, there were 21 (22%) WUSs. There were 41 males (55%) in non‐WUS group and 16 (76%) in WUS group. Mean age was quite similar in both groups (64 vs. 67) and BMI in both groups. In non‐WUS group 20% were smokers compared to 38% in WUS group. In non‐WUS group 16% have diabetes mellitus and 23% in WUS group. In non‐WUS group, 37 (50%) of patients have hypertension compared to 5 (23%) in WUS group. Atrial fibrillation was detected in 14 (19%) of non‐WUS group and 8 (38%) in WUS group. Thrombolysis rate was 10 (14%) in non‐WUS group (Table 1).

**Table 1:** Clinical characteristics of patients in non‐wake‐up stroke and wake‐up stroke group. (revised)

	**Non‐WUS**	**WUS**	
	(*n* = 74)	(*n* = 21)	***p*‐value** **
Sex, male, *n* (%)	41 (55%)	16 (76%)	0.071
Mean age, *y* (min–max)	64 (42–86)	67 (54–85)	0.314
BMI, kg m^−1^ (Hermann & Bassetti, 2009) (min–max)	28 (18–36)	29 (20–41)	0.288
Smoking, *n* (%)	15 (20%)	8 (38%)	0.150
Diabetes mellitus, *n* (%)	12 (16%)	5 (24%)	0.782
Hypertension arterialis, *n* (%)	37 (50%)	5 (24%)	0.807
Atrial fibrillation *n* (%)	14 (19%)	8 (39%)	0.459
Thrombolysis, *n* (%)	10 (14%)	0 (0%)	
Lesion location			
anterior circulation *n*(%)	58 (78%)	20 (95%)	
posterior circulation *n*	16	1	
TOAST			
1/2/3/4/5** (*n*)	16/16/20/4/18	1/8/7/0/5	
1/2/3/4/5** (%)	22/22/27/5/24	5/38/33/0/24	

	Non‐WUS	WUS	
	(*n* = 74)	(*n* = 21)	
Sex, male, *n* (%)	41 (55%)	16 (76%)	
Mean age, *y* (min–max)	64 (42–86)	67 (54–85)	
BMI, kg m^−1^ (Hermann & Bassetti, 2009) (min–max)	28 (18‐36)	29 (20‐41)	
Smoking, *n* (%)	15 (20%)	8 (38%)	
Diabetes mellitus, *n* (%)	12 (16%)	5 (24%)	
Hypertension arterialis, *n* (%)	37 (50%)	5 (24%)	
Atrial fibrillation *n* (%)	14 (19%)	8 (39%)	
Thrombolysis, *n* (%)	10 (14%)	0 (0%)	
Lesion location			
anterior circulation *n*(%)	58 (78%)	20 (95%)	
Posterior circulation *n*	16	1	
TOAST			
1/2/3/4/5* (*n*)	16/16/20/4/18	1/8/7/0/5	
1/2/3/4/5* (%)	22/22/27/5/24	5/38/33/0/24	

** Stroke etiology: 1, macroangiopathy; 2, microangiopathy; 3, cardioembolism; 4, others; 5unknown.

* Stroke etiology: 1, macroangiopathy; 2, microangiopathy; 3, cardioembolism; 4, others; 5unknown.

Included patients suffered mild‐to‐moderate neurological deficit or TIA and median NIHSS was 0 (mean 1.7) in non‐WUS and 2 (mean 3.7) in WUS group. Barthel Index was 91 in non‐WUs and 75 in WUS group. There were no statistical differences between baseline characteristics except for BI after stroke (*p* = 0.015).

**FIGURE 2:**
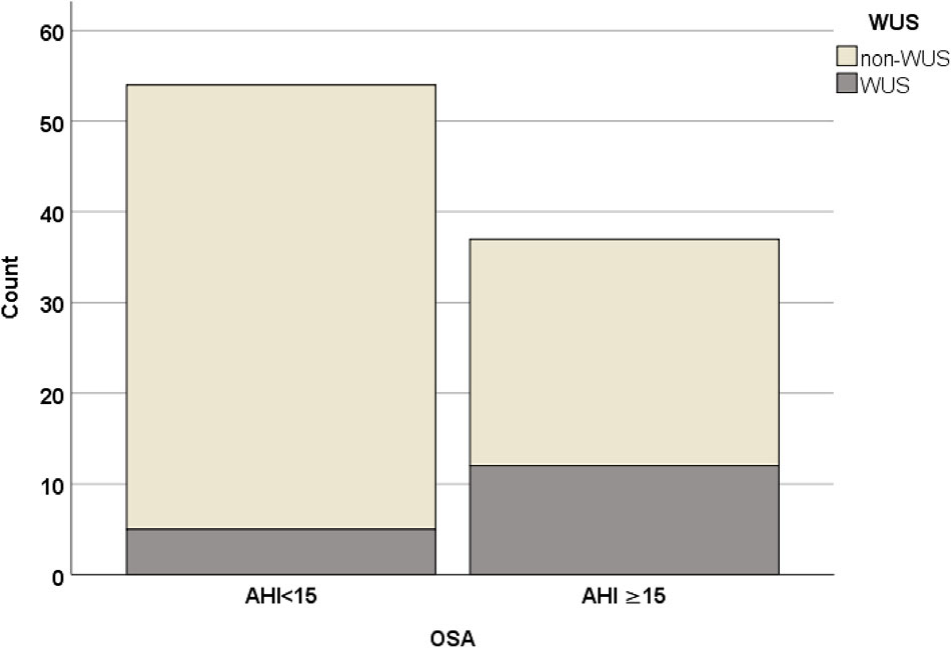
Number of non‐wake‐up stroke and wake‐up stroke patients in obstructive sleep apnea groups (AHI < 15/h and AHI > 15/h)

OSA was more frequent among WUS than non‐WUS patients (57% and 34%, respectively, *p* = 0.009). There were 12 WUS patients (32%) who had OSA (AHI > 15/h) and 5 WUS patients (11%) in non‐OSA AHI < 15/h (Figure 2).

Modified ranking scale on registration day (mRS0) was worse in WUS than non‐WUS (WUS: Median mRS0 = 2, range 0–5 and non‐WUS: Median mRS0 = 1, *p* = 0.001). At hospital discharge modified Ranking Scale (mRSexit) was still worse in WUS (WUS: Median mRSexit = 1, range 0–4 and non‐WUS: Median mRSexit = 0, *p* = 0.001) (Table 2).

**Table 2:** Comparison of patients in non‐wake‐up stroke and wake‐up stroke group

	Non‐WUS	WUS	*p*‐value^*^
AHI > 15/h, *n* (%)	25 (34%)	12 (57%)	0.009^*^
NIHSS			
Median(min–max)	0 (0–8)	2 (0–18)	
Mean (SD)	1.7 (2.4)	3.7 (4.8)	
BI			
Median(min–max)	91 (0–100)	75 (0–100)	0.015
mRS			
on registration day(mRS0) (median)	1	2	0.001^*^
At registration day (mRSexit) (median)	0	1	0.001^*^
Rehabilitation, *n* = yes (proportion)	17 (23%)	9 (43%)	0.07
Hospitalization time 5–15 days, *n* (%)	30 (41%)	11 (55%)	0.261

**FIGURE 3:**
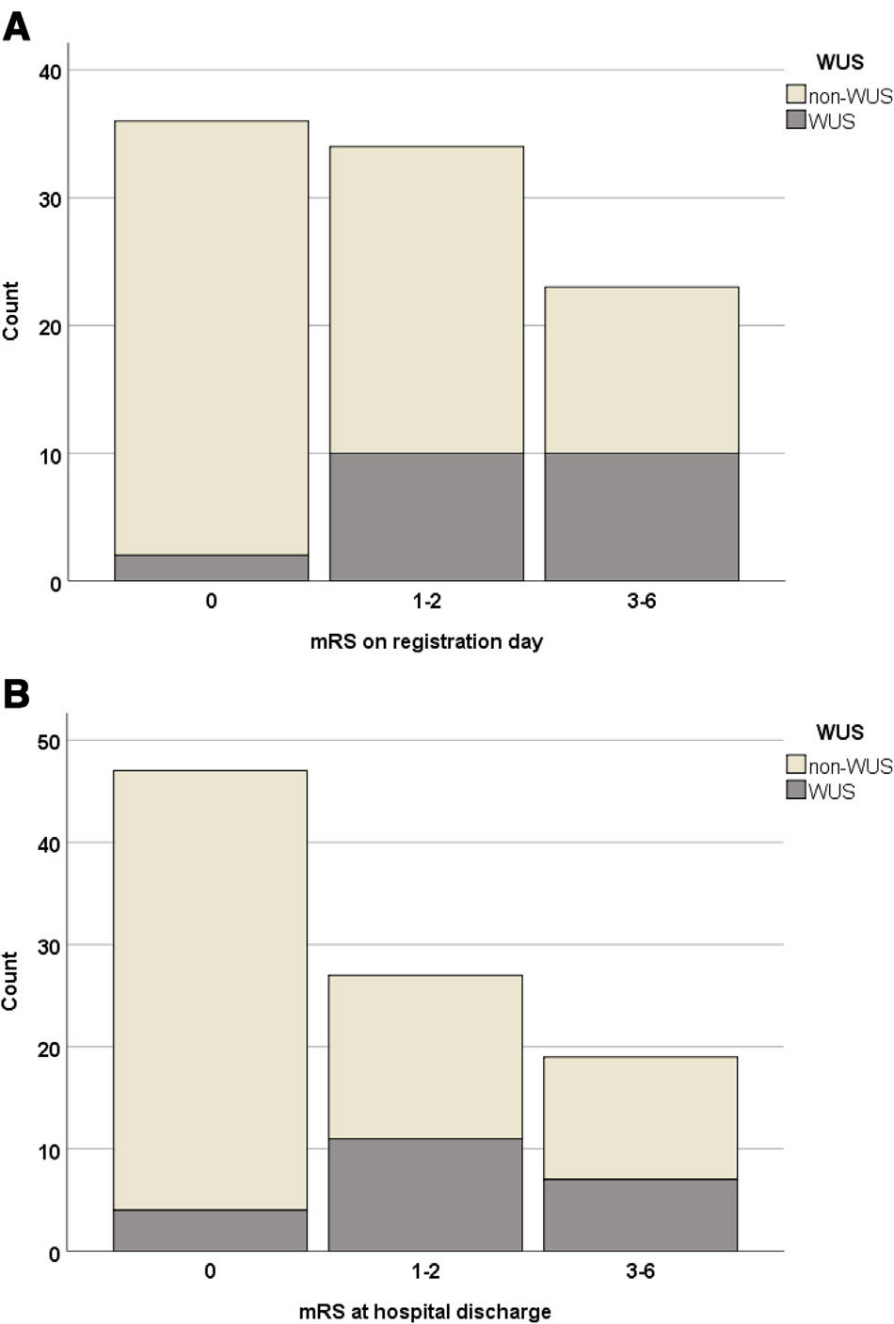
a) Number of non‐wake‐up stroke and wake‐up stroke patients in each mRS group on registration day. Proportion of wake‐up stroke patients in mRS group 1–2 and 3–6 is higher compared to non‐wake‐up stroke patients. b) Number of non‐wake‐up stroke and wake‐up stroke patients in each mRS group at hospital discharge. Proportion of wake‐up stroke patients in mRS group 1–2 and 3–6 is higher compared to non‐wake‐up stroke patients

On registration day 37 patients have mRS 0, 34 of patients had mRS 1–2 and 23 mRS 3–6. There was 1 patient with WUS (6%) in mRS 0, 10 (47%) patients with WUS in mRS 1–2 and 10 (47%) in mRS 3–6. In non‐WUS patients 36 (49%) in mRS 0, 24 (32%) in mRS 1–2, 13 (18%) in mRS 3–6 (Figure 3(a)).

At hospital discharge 48 of patients had mRS 0, 28 of patients mRS 1–2 and 19 mRS 3–6. At hospital discharge, there are 2 (10%) patients with WUS in mRS 0, 11 (52%) patients in mRS 1–2 and 8 (38%) in mRS 3–6. Of non‐WUS patients 46 (62%) in mRS 0, 17 (23%) in mRS 1–2 and 11 (15%) in mRS 3–6 (Figure 3(b)).

Patients with WUS needed rehabilitation in 43% of cases compared to 23% in non‐WUS (*p* = 0.07). Hospitalization time was more than five days in 55% of WUS and 41% of non‐WUS patients (*p* = 0.261) (Table 2).

## 4 DISCUSSION

Moderate‐to‐severe OSA and worse short‐term outcome are related to WUS in our Finnish stroke cohort. Our observations are comparable with previous studies. First, in the present study, the proportion of WUSs is 22%. Several studies have shown the prevalence of WUS to be 20–25% of all strokes (Chaturvedi et al., 1999; Elliott, 1998; Lago et al., 1998; Wroe et al., 1992).

Second, we observed a higher proportion of moderate‐to severe OSA (AHI > 15/h) in WUS compared to non‐WUS. Nearly third of OSA patients had WUS. Several studies have shown the relationship with WUS and OSA. To support our hypothesis, Iranzo et al. (2002) (Iranzo et al., 2002) concluded that night time onset were associated with the presence of moderate to severe sleep apnea (REI > 25). Bassetti et al. (2006) (Bassetti et al., 2006) found that night time stroke onset between 12:00 a.m. to 9:00 p.m. was a strong predictor of SDB. Definition of night‐time onset instead of WUS was used at the time these two studies were conducted. Two more recent studies by Hsieh et al. (2012) (Hsieh et al., 2012) and Siarnik et al. (2016) (Šiarnik et al., 2016) both found significantly higher frequency of OSA in WUS population.

Third, we found out that functional outcome measured in mRS was higher in patients with WUS compared to non‐WUS on registration day and at hospital discharge. Almost half of the patients with WUS had either mRS 1–2 or mRS 3–6 on registration day. Even if recovery was seen at hospital discharge still almost half of patients (52%) with WUS have mRS 1–2 and more than third (38%) mRS 3–6. On comparison of non‐WUS patients, majority of patients have favorable outcome, two thirds (62%) were asymptomatic and 23% have mRS 1–2 at hospital discharge. In a study by Jimenez‐Conde et al (2007), (Jiménez‐Conde et al., 2007) higher initial NIHSS score was seen in patients with WUS. In addition, they suggested a worse functional outcome and mortality at 3 months. Some studies have concluded that WUS is associated with greater initial stroke severity and worse functional outcome, (Bornstein et al., 1999; Jiménez‐Conde et al., 2007; Mackey et al., 2011; Nadeau et al., 2005) but other studies have shown no difference (Fink et al., 2002; Hsieh et al., 2012; Koo et al., 2016; Serena et al., 2003).

We could not find statistical significance in other outcome measures in the present study. There was a trend for higher need of rehabilitation among patients with WUS where almost half (43%) needed rehabilitation compared to on fourth (23%) of non‐WUS. Further, we could not find significant difference in hospitalization time. We could not find other variables related to worse short‐term functional outcome. In our previous study, higher disability and need of rehabilitation and longer hospitalization time were seen in OSA patients (Haula et al., 2020).

There are multiple mechanisms behind the link between OSA and WUS including increased platelet and fibrinogen levels (Bokinsky et al., 1995; Chin et al., 1996). In addition variable blood pressure, cerebral hypoperfusion, and increased cardiac arrhytmias (Bassetti & Aldrich, 1999; Millar‐Craig et al., 1978; Riccio et al., 2013; Siebler & Nachtmann, 1993) and reduced middle cerebral artery flow in patients with OSA has been detected (Netzer et al., 1998) which may be the possible explanations for WUS. Our results suggest that combination of OSA and WUS could predispose to stroke with higher disability and might affect stroke recovery during the first days. These findings are observed in our study even if we have mainly minor strokes and TIA and median mRS scores are low. Even though, the actual effect on outcome of WUS is controversial according to previous studies.

In our previous study, higher disability and need of rehabilitation and longer hospitalization time were seen in OSA patients. (Haula et al., 2020) Because the higher proportion of moderate‐to‐severe OSA in WUS patients we hypothesized we could find significant difference in the other two outcome measures. Reason for insignificant difference can be the low number of WUS patients with higher disability and even smaller number of those with combination of OSA and WUS in this group.

In our study majority of patients with WUS were male (76%). Koo et al. (2015) found out that men with WUS were more likely to have severe OSA. (Koo et al., 2016) Even if we could not find statistical significance in non‐WUS and WUS groups in baseline characters, the proportion of males, smokers, and patients with diabetes mellitus was higher in WUS group. These are known risk factors for OSA. We suggest that if the number of WUS patients would have been higher, it could have been statistically significant. In our previous study, higher proportion of males had OSA. In a study by Brown et al. (2018) they did not find a relationship between female, SDB, and mild WUS (Brown et al., 2018).

In the present study, proportion of WUSs in small vessel occlusion strokes was highest and second in cardioembolic strokes. In the study by Jimenez‐Conde et al. (2007), same kind of distribution of stroke subtypes was seen in WUSs (Jiménez‐Conde et al., 2007). Previous studies have shown that atherothrombotic strokes are related to WUS and OSA, but it is not seen in our cohort. According to a recent EAN/ERS/ESO/ESRS guideline by Bassetti et al. (2020) relationship between different stroke characteristics is not clearly understood (Bassetti et al., 2020).

In our study, we used AASM criteria for classifying severity of OSA, therefore the results are comparable with other similar studies. Further, the registrations in our study were done with more qualified portable wireless recorder with is approved by AASM guidelines (Berry et al., 2012). Similarly, in our study the patients were registered in acute phase within 72 h after hospital admission.

Strongest limitation of our study was that the Ethical committee did not allow major strokes to be included because of the informed consent could not be retrieved from proxy. Even though the present study includes some patients with moderate stroke who were co‐operative and communicative. In a cohort of more severe strokes, there could be more technical problems because of eventual poor co‐operation during the registration. Despite of our cohort of mainly mild strokes and TIA patients, we can compare our findings to previous international studies.

Previous studies and our present study were conducted before wake‐up trials and extended time window in stroke care, meaning the WUSs of this study did not get thrombolysis or mechanical thrombectomy. This can affect the outcome measures of wake‐up patients. Like mentioned earlier, this study included mainly TIA and minor stroke patients. Thrombolysis rate was 13.5% (*n* = 10) in this cohort. Other potential limitations of our study can be the assessment of mRS scores retrospectively from medical records. We assessed NIHSS and BI score on registration day and decided to use mRS instead of BI as an outcome measure. Modified Rankin Scale is more sensitive and useful outcome measure to show differences in mild strokes with minor neurologic deficits (Weimar et al., 2002).

*In our cohort proportion of WUS is comparable with previous studies (22%) and proportion of moderate‐to‐severe OSA is 42%. OSA was more common in WUS patients (57%) than in non‐WUS (34%). Thus, our study raises question if screening of OSA be should be used routinely for preventing WUS and possible strokes with higher disability. However, resources do not allow all stroke or TIA patients to be tested with polysomnography or respiratory polygraphy. Therefore, the need for prescreening tools exits. Based on our findings, no causal relationship can be established between WUS, OSA, and worse functional outcome. Even though, based on our findings, a clinician may consider screening of OSA with respiratory polygraphy in WUS patients*.

## 5 CONCLUSION

We conclude that the proportion of moderate to severe OSA is higher in stroke and TIA patients with WUS compared to patients with non‐WUS. In addition, patients with WUS have worse short‐term functional outcome measured in mRS during hospitalization. Further studies are needed to determine if OSA is causally linked to WUS.

## FUNDING INFORMATION

The research was supported by the State Research Funding (Southwest Hospital District, Finland).

### PEER REVIEW

The peer review history for this article is available at https://publons.com/publon/10.1002/brb3.2284.

The approval number of Ethics Committee of the Hospital District of Southwest Finland: ETMK:54/180/2012.

Data registry holder (in Finnish): https://www.satasairaala.fi/tutkimus/satakunnan‐sairaanhoitopiirin‐tutkimusluvat

## Data Availability

Data available on request. Data includes sensitive data. Ethic committee statement for data usage is needed from the Ethics Committee of the Hospital District of Southwest Finland as well as permission to use data from Data register holder, Hospital District of Satakunta. Ethics committee: http://www.vsshp.fi/en/tutkijoille/eettinen‐toimikunta/Pages/default.aspx
